# A novel *COMP *mutation in a pseudoachondroplasia family of Chinese origin

**DOI:** 10.1186/1471-2350-12-72

**Published:** 2011-05-21

**Authors:** Li Dai, Liang Xie, Yanping Wang, Meng Mao, Nana Li, Jun Zhu, Christopher Kim, Yawei Zhang

**Affiliations:** 1 National Center for Birth Defects Monitoring, West China Second University Hospital, Sichuan University, 20 Ren Min Nan Lu Section 3, Chengdu 610041, China; 2 Department of Pediatrics, West China Second University Hospital, Sichuan University, 20 Ren Min Nan Lu Section 3, Chengdu 610041, China; 3 National office for Maternal and Child Health Surveillance, West China Second University Hospital, Sichuan University, 20 Ren Min Nan Lu Section 3, Chengdu 610041, China; 4 Yale University School of Public Health, 60 College street, New Haven, CT 06520, USA

## Abstract

**Background:**

Pseudoachondroplasia (PSACH) is caused exclusively by mutations in the gene for cartilage oligomeric matrix protein (*COMP*). Only a small number of studies have documented the clinical phenotype and genetic basis in Chinese PSACH patients.

**Case presentation:**

We investigated a four-generation PSACH pedigree of Chinese Han origin. Two patients and two unaffected individuals were recruited for clinical evaluation and molecular genetic analysis. The genomic DNA was extracted from peripheral blood leukocytes. Polymerase chain reaction (PCR) was adopted to amplify the 8-19 exons of *COMP *gene. Then the products were sequenced bi-directionally for screening mutation. Clinical evaluation revealed that PSACH patients in this pedigree had a severe disproportionate short stature (-10SD). A heterozygous TGTCCCTGG insertion in exon 13, between nucleotide 1352T and 1353G, were identified in the patients except the unaffected individuals, which resulted in a three-amino-acid insertion (451V_452P ins VPG) in the sixth calmodulin-like repeat of the *COMP *protein.

**Conclusion:**

This c. 1352_1353ins TGTCCCTGG is a novel mutation responsible for severe familial PSACH.

## Background

Pseudoachondroplasia (PSACH, OMIM 177170) is a rare autosomal dominant osteochondrodysplasia characterized by typical disproportionate short stature, brachydactyly, lower limbs anomalies, joint laxity, scoliosis, early onset osteoarthritis, epiphyseal and metaphyseal abnormalities. It is estimated to affect at least 1 in 20,000 persons [1,2]. The affected individual has a normal length at birth,and growth retardation generally will not be recognized until two or three years of age. Notably, all patients have normal craniofacial appearance and intelligence [1,3-5].

The PSACH gene was initially localized to chromosome 19 in 1993 [6,7]. Mutations on the Cartilage Oligomeric Matrix Protein (*COMP*) gene were subsequently found to cause PSACH and another skeletal dysplasia, multiple epiphyseal dysplasia [8,9]. COMP protein is a large secreted pentameric glycoprotein of the thrombospondin family, expressed in the extracellular matrix (ECM) surrounding the cells that make up ligaments and tendons. COMP monomer consists of an amino-terminal domain, four type IIepidermal growth like repeats, eight type III calmodulin-like repeats (CLRs), and a carboxyl-terminal globular domain. Numerous mutations of *COMP *gene have been found to date, most of them occur in the CLR regions (~85%), while others in the C-terminal globular domain (~15%) (the Human Gene Mutation Database, http://www.hgmd.cf.ac.uk/ac/index.php). Although the normal function is not fully known, the pathological link between gene mutation, protein alteration, abnormal cell growth and PSACH has been established [1,10-17]. More than 60 *COMP *mutations have been identified to be with PSACH [10,18], but only a few in Chinese patients [19,20]. Severe short stature (-6 SD) in PSACH has been associated with mutations in exon 13, particularly in the region encoding CLR7 of *COMP *protein [21]. Elliott AM et al recently detected the c.812A→T in exon 8 in a sporadic Inuit PSACH patient with severe short stature (-7.5 SD) [22]. In the present study, we examined the *COMP *gene for mutations in a Chinese family affected by severe PSACH.

## Case presentation

### Subjects and methods

**Figure 1:**
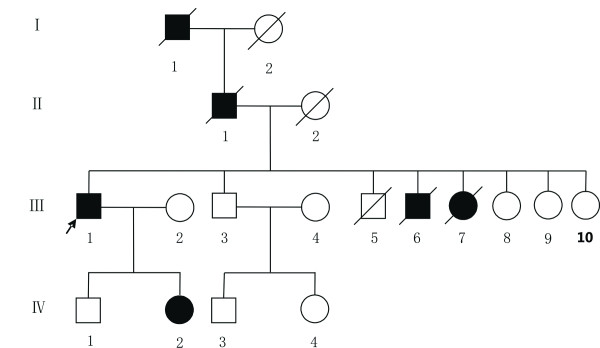
**Pedigree chart of a Han Chinese pesudoachoplasia family**.

This family was initially contacted in the Sichuan province through a member hospital in the Chinese Birth Defects Monitoring Network. PSACH disorder was transmitted through at least four generations in this family. (Figure 1) After informed consent, two affected (III 1 and IV 2) and two healthy persons (III 3 and IV 4) received a comprehensive physical examination and had blood samples taken for mutation screening. The clinical records and radiographic images were published under the patients' written permission. This study was approved by the Research Ethics Committee of Sichuan University.

Using a commercially available kit (Bio Teke, Beijing, China), genomic DNA was isolated from blood samples utilizing the standard protocol provided by the manufacturer of the kit. The procedure used for amplifying *COMP *exons 8-19 (accession number NM000095) and PCR is outlined previously [23]. The resulting product was purified, separated by PAGE, and sequenced bi-directionally using an ABI 3730xl sequencer.

### Clinical features and skeletal radiographic findings

**Figure 2:**
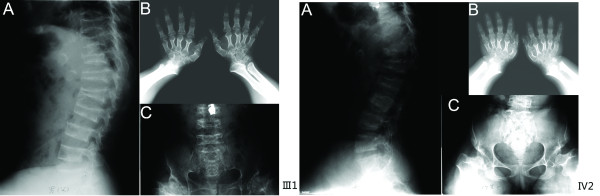
**Similar radiographic features for individual III1 and IV2**. (A) Anterior beaking of the vertebral bodies and distinctive plantyspondyly, mild lumber lordosis. (B) Short metacarpals and phalanges, small carpal bones, cone-shaped epiphyses, irregular metaphyses of the ulna and radius. (C) Mild scoliosis, small femoral heads, flared metaphyseal borders, poorly developed acetabulae with enlarged acetabular angles, and widened symphysis pubis.

Pedigree analysis revealed six individuals affected by severe short stature within a span of four generations and the disorder exhibited dominant autosomal transmission. All affected individuals demonstrated the typical characteristics of PSACH: disproportionate short-limb dwarfing, short upper limbs, lower limb abnormities, waddling gait, but normal facial features and intelligence. The abnormalities of the affected members in this family appeared at about one year of age, usually presenting as difficulties in learning to walk and delayed growth compared with normal children. The proband (III 1, Figure 1) was 99.6 cm tall at 45 years of age (-10.6 SD on a normal growth curve of rural Chinese male adults [24]; -1 SD on PSACH curve [25]), less than the average height of 101.8 cm of typical rural-born 4-year-old boys [24]. He had strikingly short limbs (upper limb length 41.2 cm), small hands, short fingers, crus varum and waddling gait. A lateral spine radiograph (Figure 2) indicated platyspondyly and anterior beaking of the vertebrae. His phalanges and metacarpals were short with cone-shaped epiphyses. (Figure 2) The AP view of pelvis radiograph (Figure 2) showed small femoral heads, irregular acetabulae, enlarged acetabular angles and widened symphysis pubis. His affected daughter (IV2, Figure 1) demonstrated similar characteristics, (Figure 2) albeit somewhat more severe than her father. She was only 96 cm tall (-11.2 SD on a normal growth curve of rural Chinese females at 16 years [26]; -1 SD on PSACH curve [24]) when was referred to hospital at 16 years of age, which is comparable to the height of a 3-year-old rural-born girl, far shorter than the 156 cm average height of 16-year-old rural-born girls [24]. The proband's brother (III6, Figure 1, died two years ago) was afflicted by a shorter stature.

### Mutational analysis of the COMP gene

**Figure 3:**
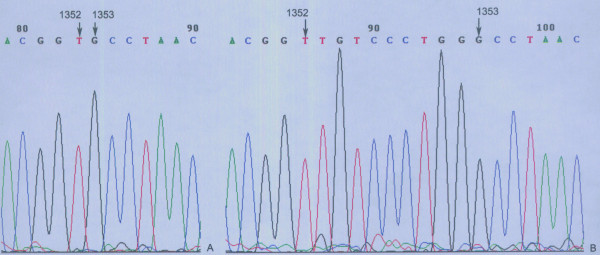
**The mutation, c.1352_1353insTGTCCCTGG, in exon 13 of *COMP *gene**. (A) Normal DNA fragment encoding animo acids Thr-Val-Pro-Asn; (B) mutant sequence encoding animo acids Thr-Val-Val-Pro-Gly-Pro-Asn.

After sequencing the *COMP *exons 8-19 in the two patients and two unaffected individuals, a heterozygous 9 bp insertion (c.1352_1353insTGTCCCTGG) was identified between nucleotide 1352T and 1353G in exon13, which is located at the end of the sixth repeat of the type-3 calcium-like repeat region of the *COMP *gene. (Figure 3) As a consequence, three additional amino acids, valine, proline and glycine, were inserted between residues 451V and 452P of the *COMP *protein (p.451V_452PinsVPG). A recent analysis indicated that the residues 451V and 452P are in a calcium-binding pocket domain of *COMP *protein [27]. The result was confirmed by repeat sequence analysis.

## Conclusion

Up to now, *COMP *is the only gene known to be associated with pseudoachondroplasia. *COMP *mutations and pathological consequences have been extensively studied in Caucasians, but a few in the Chinese population. Besides the c.815C→T in exon 8 [19] and the c.1447-1455del [20] in exon 13 of *COMP *gene reported lately in Chinese PSACH patients, we identified the c. 1352_1353insTGTCCCTGG within exon 13 of *COMP *that contributes to severe PSACH phenotype in a Han Chinese pedigree. This novel trinucleotide expansion was located in the region coding the sixth CLR (p. 451V_452PinsVPG) and expands the knowledge of *COMP *mutations leading to PSACH.

PSACH mutations are located in exons 8-14 of *COMP *gene encoding the type III CLRs of COMP protein [3,18,28]. The most frequent mutation, present in one third of PSACH patients, is the deletion of GAC (c.1405-1419delGAC) in a very short triplet repeat (GAC5), which encodes five consecutive aspartic acid residues within the CLR7 of the protein, usually referred as p.469delD, p.473 delD or p.del D469-473 [3,8,29,30]. Most other *COMP *mutations involve the substitution of one amino acid for another in the *COMP *protein. Trinucleotide expansion at the region encoding type III CLRs of the protein has now been reported in this study and one other previous study [31]. Genotype-phenotype studies revealed that mutations in CLR7 were associated with more severe PSACH phenotypes than those in elsewhere in the CLRs [21]. The early onset characteristics of PSACH, severe short stature, generalized dysplasia of epiphyses and metaphyses, in these individuals with mutations in other CLRs continues to enforce the importance of the *COMP *gene.

COMP is a multifunctional structural protein that affects cellular attachment, proliferation and chondrogenesis. It also participates in the ECM assembly through interactions with numerous proteins including types I, II, IV, XI, XII collagen, decorin, fibronectin and matrilin-3 [32,33]. According to the newly published crystal structure of the *COMP *protein [27], the p.451V_452PinsVPG was located to a pocket structure in the end of the sixth CLR of the *COMP *protein, where a calcium ion is embedded. Calcium supports the binding of COMP to fibronectin which reportedly influences the conformation of COMP [32]. Specifically, alterations in CLRs make calcium-dependant protein folding malfunction [11,30,32,34-37], lead to the assembly of mutant *COMP *and other proteins in the rER of affected chondrocytes [33]. This intracellular retention is cytotoxic and prevents efficient secretion of these proteins, alters the extracellular matrix and results in premature chondrocyte death. Eventually, the upregulated apoptosis of chondrocytes from the growth plate diminishes linear growth, and the abnormal ECM causes early onset osteoarthritis observed in PSACH patients [1,38-40]. In PSACH, only a small fraction of COMP pentamers (3% or less) contains all wild-type COMP subunits, most of the COMP pentamers contain one or more mutant subunits that exert dominant negative effects [8,30]. A mouse model built by Posey et al. recapitulated this cellular pathology of human PSACH, including the retention of ECM proteins, intracellular matrix formation in the rER cisternae, and increased apoptosis of chondrocyte [11]. Previous studies have shown that calcium-binding is critical in the function of the *COMP *protein, and missense mutations or in-frame deletions in the calmodulin-like repeats region reduces this ability [1]. The longer amino acids sequence in CLR6 due to this novel mutation may have similar negative effects on the function of the *COMP *protein, the proliferation and apoptosis of chondrocytes. This study suggests that the 9 bp insertion can be responsible for the severe phenotype in the Chinese family in despite no direct functional evidence. In conclusion, the c.1352_1352insTGTCCCTGG within exon 13 of *COMP *expands the spectrum of mutations that causes PSACH. It could be of value to the molecular diagnosis, genotype-phenotype relationship understanding and pathogenesis of this disorder.

## Abbreviations

PSACH: pseudoachondroplasia; COMP: cartilage oligomeric matrix protein; CLR: calmodulin-like repeat domain; ER: endoplasmic reticulum; ECM: extracellular matrix.

## Competing interests

The authors declare that they have no competing interests.

## Authors' contributions

LD studied the family, designed research plan and prepared manuscript. LX and NL performed the molecular genetic studies, participated in sequence alignment. YW and MM participated in clinical evaluation. JZ participated in collecting specimens and supervised study. CK and YZ reviewed and revised the manuscript. All authors had read the final manuscript and approved the publication of the clinical images.

## Pre-publication history

The pre-publication history for this paper can be accessed here:

http://www.biomedcentral.com/1471-2350/12/72/prepub
